# Effect of Nano-Selenium on Intestinal Oxidative Stress Induced by H_2_O_2_ in Mice

**DOI:** 10.3390/antiox14091073

**Published:** 2025-09-01

**Authors:** Xiangyu Mao, Wenyuan Li, Yuanyuan Li, Xuemei Jiang, Ruinan Zhang, Lianqiang Che, Yong Zhuo, Mengmeng Sun, Xianxiang Wang, De Wu, Shengyu Xu

**Affiliations:** 1 Animal Disease-Resistance Nutrition, Ministry of Education, Ministry of Agriculture and Rural Affairs, Key Laboratory of Sichuan Province, Animal Nutrition Institute, Sichuan Agricultural University, Chengdu 611130, China; mxy010117@163.com (X.M.); liwenyuan0106@163.com (W.L.); perseverance_bai@163.com (Y.L.); 71310@sicau.edu.cn (X.J.); 72022@sicau.edu.cn (R.Z.); che.lianqiang@sicau.edu.cn (L.C.); zhuoyong@sicau.edu.cn (Y.Z.); wude@sicau.edu.cn (D.W.); 2 College of Science, Sichuan Agricultural University, Xin Kang Road, Yucheng District, Ya’an 625014, China; sunmeng14391@163.com; 3 College of Science, Sichuan Agricultural University, Chengdu 611130, China; xianxiangwang@hotmail.com

**Keywords:** nano-selenium, intestine, oxidative stress, antioxidant capacity, selenoprotein

## Abstract

Selenium is an important trace element with certain antioxidant effects. Nano-selenium, as a novel selenium source, has the advantages of strong biological activity, high absorption efficiency, and low toxicity. The aim of the present study was to compare the protective effects of sodium selenite and nano-selenium on intestinal oxidative stress induced by hydrogen peroxide (H_2_O_2_) in mice. A total of 60 female mice were randomly divided into 6 groups with 10 replicates per group and 1 mouse per replicate (*n* = 10). The first three groups were as follows: the Control group (C), fed with basal diet; the sodium selenite group (SS), basal diet + 0.3 mg·kg^−1^ sodium selenite; and the nano-selenium group (NS), basal diet + 0.3 mg·kg^−1^ nano-selenium. The latter three groups (CH, SSH, NSH) were fed the same diet as the former three groups, but the last 10 days of the experiment were fed with drinking water containing 0.3% H_2_O_2_ to induce oxidative stress. The results showed that under normal conditions, the supplementation with sodium selenite or nano-selenium decreased the spleen index of mice; sodium selenate up-regulates *GPX3* expression in the ileum, and increases T-SOD in the colon of mice; and nano-selenium up-regulated *GPX1* expression but decreased T-AOC in the jejunum. After drinking water treated with H_2_O_2_, H_2_O_2_ increased the expression of intestinal inflammatory factors and selenium proteins, such as *IL-1β* and *SOD* in jejunum, *IL-1β*, *NF-κB*, *IL-10*, *TXNRD1*, *TXNRD2*, *GPX1*, *GPX3*, *GPX4*, and *CAT* in ileum, and *IL-1β* and *SOD* in colon. At the antioxidant level, H_2_O_2_ decreased T-AOC in the jejunum. In the H_2_O_2_ treatment, sodium selenite and nano-selenium increased the ratio of VH to CD (VH/CD) in jejunum; sodium selenite up-regulated the expression of *TXNRD1* in jejunum, down-regulated the expression of *GPX3* in ileum, at the antioxidant level, decreased the T-SOD and T-AOC in colon, and increased the content of MDA in ileum; and nano-selenium down-regulated the expression of *TXNRD1* in colon. At the same time, the expression of *IL-1β*, *NF-κB*, *IL-10*, *TXNRD1*, *TXNRD2*, *GPX1*, *GPX4*, and *CAT* can be restored to normal levels by selenium supplementation. According to the results, drinking H_2_O_2_ induced intestinal oxidative stress in mice to a certain extent, and selenium supplementation mitigated the destructive effect of H_2_O_2_ on the intestinal morphology of mice jejunum and restored the level of related inflammatory factors, and had a positive effect on antioxidants.

## 1. Introduction

The intestinal tract is essential for nutrient digestion and absorption in animals. It also serves as a primary immune barrier and a major site of reactive oxygen species (ROS) production. Small and moderate amounts of ROS contribute to killing invading pathogens, wound healing, and tissue repair, as excessive ROS can cause tissue oxidative damage [1]. Oxidative stress is defined as an imbalance between the amount of reactive oxygen species/nitrogen species (ROS/RNS) produced and the ability of cells to neutralize them through antioxidant defenses [2]. Therefore, the intestinal tract is susceptible to exogenous and metabolic oxidative free radical damage, inducing intestinal oxidative stress, which may lead to a variety of intestinal diseases and cause huge losses to animal husbandry.

Selenium is an essential trace mineral element for animals, and its organic form is mainly selenocystine. Selenoprotein is formed through this organic form and plays an important role in many biological functions, such as anti-oxidation in the body, the formation of thyroid hormones, DNA synthesis, and positive effects on fertility and reproduction [3]. At the same time, selenium also plays a positive role in anti-inflammation, improving immune function, and promoting animal growth [3,4]. Nanotechnology refers to a comprehensive technical system for the preparation and research of substances at the nanoscale. Compared with single atoms or other large particles, nanoparticles exhibit new characteristics, such as larger surface area and smaller particle size, large specific surface area, high catalytic activity, good uniformity, and good physical reaction [5,6]. The novel selenium source nano-selenium is a nano-scale pink powder, which has the properties of nanoparticles and shows the advantages of high biological activity, high bioavailability, high absorption rate, low toxicity, high safety, and chemical stability. It also has a variety of biological activities, such as immune system, antioxidant, antiviral, and anti-cancer activities [7,8].

Therefore, it is of great significance to study whether nano-selenium has a better protective effect in animals. At present, there have been many studies and applications of nano-selenium. In the rat model of L-arginine-induced acute pancreatitis, through the analysis of histopathology, related pancreatic enzymes, antioxidant enzymes, inflammatory factors, immune factors, apoptosis factors, NF-κB, and other factors, the supplementation of nano-selenium has a positive effect on the endocrine and exocrine functions of acute pancreatitis [9]. Kondaparthi et al. [10] used different doses of nano-selenium and sodium selenite to study the liver of mice, and found that low doses of nano-selenium had better antioxidant effects in the liver. As for the effect of nano-selenium on the animal digestive tract, it has been found that nano-selenium has positive effects on intestinal homeostasis and mucosal immune defense by regulating goblet cells in laying hens [11]. Liu et al. [12] studied the protective effect of nano-selenium on intestinal injury induced by a high-fat diet (HFD) in young grass fish. They analyzed intestinal morphology, tight junction, inflammation, antioxidants, and intestinal flora, and the results showed that nano-selenium supplementation reduces intestinal injury caused by HFD and maintains the integrity of the intestine. Meanwhile, relevant research has been conducted on the nano-selenium (SENP) used in the present study. They found that the nano-selenium alleviates the immune response and apoptosis of lipopolysaccharide (LPS)-induced yak testicular interstitial cells by regulating the expression of genes related to apoptosis and inflammation [13].

At present, there are few studies on the relationship between nano-selenium and the intestinal tract, and few studies on the prevention and protection of different selenium sources on mammalian intestinal oxidative damage. Therefore, in the present study, mice were fed with 0.3% H_2_O_2_ in drinking water to induce oxidative damage, and the related indicators were measured to analyze the effects of nano-selenium on H_2_O_2_-induced intestinal oxidative stress.

## 2. Materials and Methods

### 2.1. Preparation of Chitosan Nano-Selenium Composites

Initially, chitosan nano-selenium (CS-SeNPs) was synthesized by dissolving 1.0 g of chitosan powder in a 1% (*w*/*w*) acetic acid solution. This mixture was stirred continuously at room temperature for 6–12 h to completely dissolve, yielding a clarified, slightly pale-yellow solution at a concentration of 10 mg/mL, which was set aside as a reserve liquid. Subsequently, 1 mL of a 50 mM Na_2_SeO_3_ solution and 0.08 mL of the earlier prepared chitosan reserve liquid were briskly transferred into a beaker and promptly diluted to 8 mL with distilled water. With continuous stirring and shaking, 2 mL of a 100 mM ascorbic acid (Vc) solution was gradually added dropwise. The color transition of the mixture from colorless to bright red indicated the formation of CS-SeNPs. Following formation, the colloidal mixture was loaded into a dialysis bag with a molecular weight cut-off of 3500 and subjected to dialysis for 24 h, with changes in distilled water every 5–8 h. This process yielded CS-SeNPs at a concentration of 5 mM. The nano-selenium composites were then stored at 4 °C for further use.

### 2.2. Mice and Management

**Figure 1:**
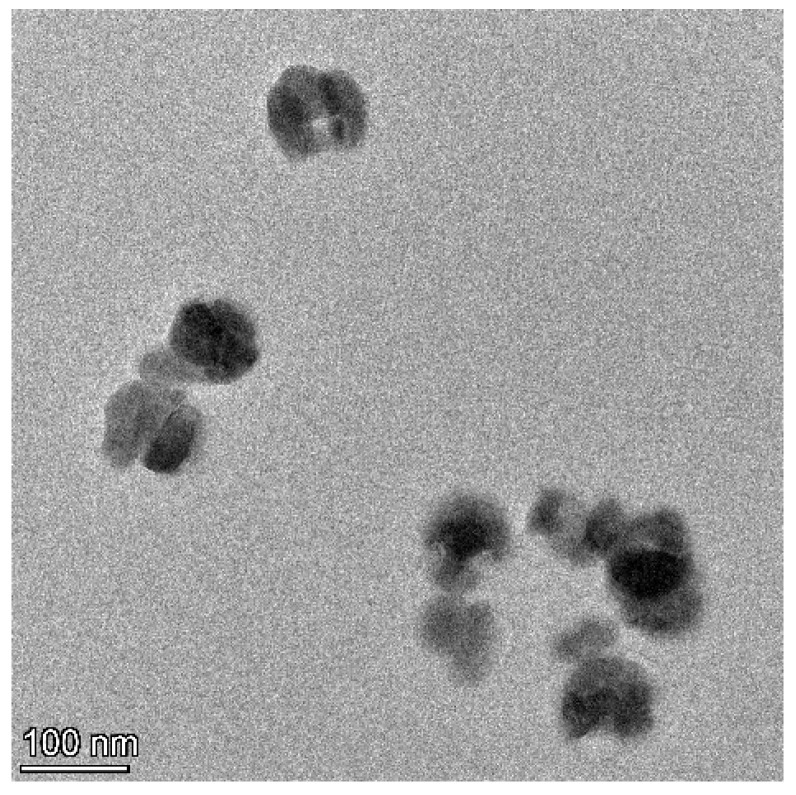
Transmission electron microscope image of nano-selenium.

**Figure 2:**
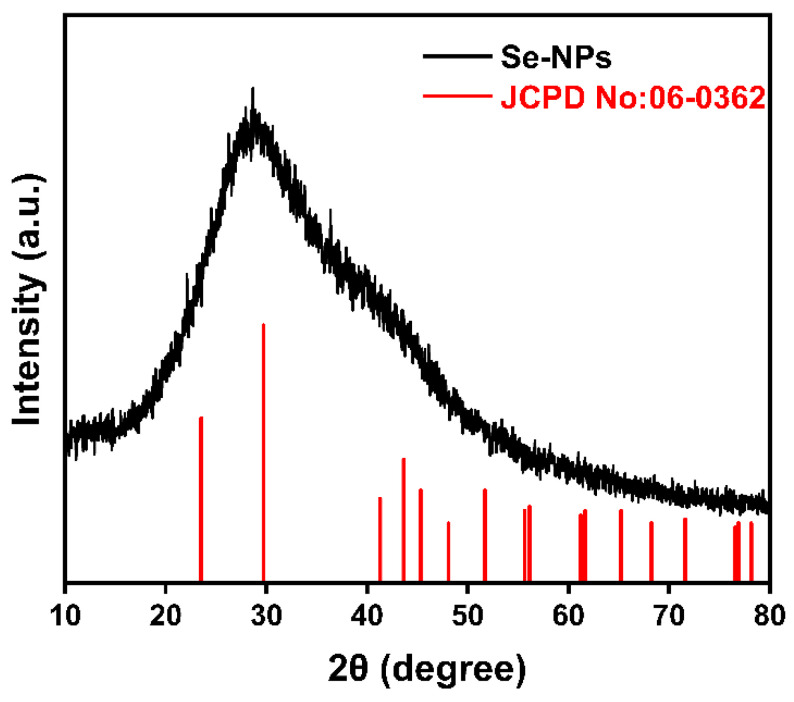
X-ray diffraction patterns of nano-selenium.

Sixty 3-week-old specific pathogen-free (SPF) female mice (Institute of Cancer, ICR) were randomly divided into 6 groups with 10 replicates per group and 1 mouse per replicate (*n* = 10). The 6 groups were (1) Control (C): basal diet; (2) sodium selenite group (SS): basal diet + 0.3 mg·kg^−1^ sodium selenite; (3) nano-selenium group (NS): basal diet + 0.3 mg·kg^−1^ nano-selenium; (4) Control + H_2_O_2_ group (CH): basal diet + 0.3% H_2_O_2_ drinking water; (5) sodium selenite + H_2_O_2_ group (SSH): basal diet + 0.3 mg·kg^−1^ sodium selenite + 0.3% H_2_O_2_ drinking water; and (6) nano-selenium + H_2_O_2_ group (NSH): basal diet + 0.3 mg·kg^−1^ nano-selenium + 0.3% H_2_O_2_ drinking water. Sodium selenium was obtained from Chengdu Shuxing Feed Co., Ltd., Chengdu, China. Nano-selenium was graciously provided by Professor Xianxiang Wang at the College of Science, Sichuan Agricultural University. The nano-selenium exhibits a spherical morphology, with a uniform particle size distribution of approximately 50 nanometers (Figure 1). The powder X-ray diffraction (XRD) pattern of the nano-selenium reveals the presence of monoclinic Se8 crystalline domains; however, the crystallinity is not high enough to produce distinct peaks (Figure 2). The test period was 8 weeks and 10 days. The mice in groups 4, 5, and 6 were allowed to drink ad libitum with 0.3% H_2_O_2_ water for the last 10 days. During the experiment, the mice were provided with sufficient drinking water and feed every day, given free access to feed and water, the cages were changed every 5–7 days, and clean bedding was provided. The laboratory was ventilated, clean, and quiet; the ambient temperature was maintained at 23 °C, and the dark/light cycle was 12 h. The formula of the basic diet is shown in Table 1. In order to add selenium to the basic diet, the sodium selenite group and the selenium nanoparticles group were fully stirred in the prepared feed to ensure that all the ingredients in the feed were fully mixed.

### 2.3. Determination of Selenium Content in Feed

According to the People’s Republic of China National Standard GB/T 13883-2023 [14], the main steps include: (1) acid digestion, (2) dilution of the digestion fluid, (3) preparation of the standard curve, and (4) measurement. The specific operation was carried out according to GB/T 13883-2023.

The basal dietary selenium content was determined to be 0.042 mg·kg^−1^.

### 2.4. Sampling

On the last day of the experiment, the mice were fasted overnight and sacrificed by CO_2_ after recording their living weight. The liver, spleen, kidney, heart, and pancreas were dissected and weighed, and the organ index was calculated according to the living weight. Organ index = organ weight/live weight.

After the mice were dissected, the whole jejunum, ileum, and colon tissues were collected and washed with normal saline. About 1 cm of the middle segment of the jejunum was taken and placed in 10% paraformaldehyde for intestinal morphological analysis. The remaining jejunum, ileum, and colon were placed in 2 mL cryotubes and snap-frozen in liquid nitrogen tanks, then stored at −80 °C for subsequent determination of antioxidant enzyme and related inflammatory factor gene expression and antioxidant indices.

### 2.5. Intestinal Histomorphology

The collected jejunal tissues were wrapped in paraffin. Paraffin sections were sectioned into 5–6 μm thick pieces using a microtome and stained with hematoxylin and eosin (H&E). The villi height (VH) and crypt depth (CD) of the jejunum were measured by NDP.view 2.9.22, and the ratio of VH to CD (VH/CD) was calculated. Ten complete and longest intestinal villi were selected from each section to measure their height, and the depth was determined by selecting the root of or near the crypt of the measured intestinal villi.

### 2.6. Measurement of Expression Levels of Antioxidant Enzymes and Inflammatory Factors in Intestinal Tissues

#### 2.6.1. Total RNA Isolation

Trizol was added to the tissue samples to extract total RNA. Then, chloroform shaking and centrifugation were performed. Followed by isopropanol addition for RNA precipitation and washing. The RNA precipitate was completely dissolved in an appropriate amount of diethylpyrocarbonate water (DEPC water). After dissolution, the RNA concentration was determined, and then reverse transcription was performed immediately.

#### 2.6.2. Reverse Transcription

RNA was reverse transcribed to cDNA using the Novizan Bio HiScript^®^ III RT SuperMix for qPCR (+gDNA wiper) kit (R323-01, Novizan Biotech Co., Ltd., Nanjing, China). Methods and procedures were performed according to the instructions.

#### 2.6.3. Real-Time Fluorescent Quantitative PCR

The ChamQ Universal SYBR qPCR Master Mix kit (Q711-02, Novizan Biotech Co., Ltd., Nanjing, China) was used for the assay. Amplification procedures and method steps were performed according to the instructions. Relative gene expression was calculated using the 2^−ΔΔCt^ method [15].

#### 2.6.4. Measurement Indicators

Inflammatory factors in the jejunum, ileum, and colon included interleukin-1β (*IL-1β*), tumor necrosis factor (*TNF-α*), nuclear factor kappa B (*NF-κB*), and interleukin-10 (*IL-10*).

Selenoproteins and antioxidant enzymes in jejunum, ileum, and colon tissues included thioredoxin reductase 1 (*TXNRD1*), thioredoxin reductase 2 (*TXNRD2*), glutathione peroxidase 1 (*GPX1*), glutathione peroxidase 2 (*GPX2*), glutathione peroxidase 3 (*GPX3*), glutathione peroxidase 4 (*GPX4*), superoxide dismutase (*SOD*), and catalase (*CAT*).

The primer sequences used in the PCR experiments were retrieved from the National Center for Biotechnology Information website and synthesized at Beijing Tsingke Biotech Co., Ltd., Beijing, China. The primer sequences are shown in Table 2.

### 2.7. Measurement of Antioxidant Indicators

Appropriate amounts of jejunum, ileum, and colon tissue were weighed, washed, and homogenized in physiological saline. The samples were diluted 10 times with physiological saline according to the weight of the samples in a frozen storage tube and homogenized in a homogenizer by adding steel beads. We transferred the homogenate to a centrifuge tube. Centrifugation was performed at 3000 r·min^−1^ for 15 min. The supernatant was collected, and the protein concentration was determined by Beyotime P0010 BCA Protein Concentration Assay kit (enhanced) (P0010S, Beyotime, Shanghai, China). According to the protein concentration, the activities of superoxide dismutase (SOD, A001-1-2), total antioxidant capacity (T-AOC, A015-2-1), catalase (CAT, A007-1-1), and malondialdehyde (MDA, A003-1-2) in the jejunum of mice were measured by antioxidant kits purchased from Nanjing Jiancheng Bioengineering Institute (Nanjing, China). T-AOC and MDA content in the ileum were measured. The contents of SOD, T-AOC, and MDA in the colon were measured. The method and steps of the determination were strictly in accordance with the instructions in the kit.

### 2.8. Statistical Analysis

All data were preliminarily sorted using Excel 2020, and then a *t*-test was performed between normal drinking water and H_2_O_2_ treatment using SAS 9.4 software. One-way ANOVA was used between C, SS, NS, and CH, SSH, NSH, and multiple comparisons were made by the Tukey method. Data were expressed as mean ± standard error, *p* < 0.05 as a significant level, 0.05 ≤ *p* ≤ 0.1 as a trend. At the same time, SAS 9.4 software was used to calculate the effect size, and the effect size was calculated in one-way ANOVA: eta-squared (*η^2^*), *η^2^* < 0.06 was a minor effect, 0.06 ≤ *η^2^* < 0.14 was a medium effect, and *η^2^* ≥ 0.14 was a large effect. Effect sizes were calculated in the *t*-test as Hedges’ g (*g*), with |*g*| < 0.5 as a minor effect, 0.5 ≤ |*g*| < 0.8 as a medium effect, and |*g*| ≥ 0.8 as a large effect.

## 3. Results

### 3.1. Effect of Nano-Selenium on Organ Index

Under normal conditions, the group SS and group NS significantly reduced the spleen index of mice, and the effect size was large (*p* < 0.05, *η^2^* = 0.219, 95% CI [0.036,0.52], Table 3). However, for the kidney index, selenium supplementation was not significant, but the effect size was large (*p* > 0.05, *η^2^* = 0.147, 95% CI [0.016,0.50]). Compared with group NS, group NSH significantly increased the spleen index and the effect size was large (*p* < 0.05, *g* = −1.011, 95% CI [−2.00,−0.02]), and there was no significant difference in the kidney index between the two groups, but the effect size was large (*p* = 0.05, *g* = 0.891, 95% CI [−0.06,1.84]). However, there was no significant effect on the organ indices of liver, kidney, heart, and pancreas between nano-selenium and sodium selenite under normal conditions or H_2_O_2_ oxidative stress treatment, and the effect size is small or medium (*p* > 0.05, *η^2^* < 0.14, |*g*| < 0.8).

### 3.2. Effect of Nano-Selenium on the Morphology of Jejunum

**Figure 3:**
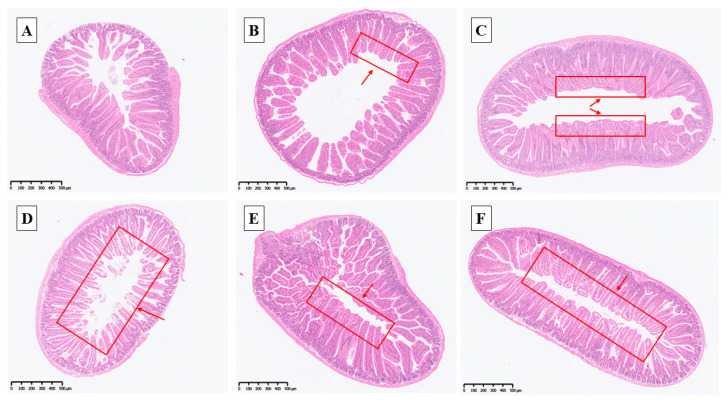
Diagram of jejunum sections in each group (HE staining, magnification: 50×). (**A**) Control: basal diet; (**B**) sodium selenite group: basal diet + 0.3 mg·kg^−1^ sodium selenium; (**C**) nano-selenium group: basal diet + 0.3 mg·kg^−1^ nano-selenium; (**D**) Control + H_2_O_2_ group: basal diet + 0.3% H_2_O_2_ drinking water; (**E**) sodium selenite + H_2_O_2_ group: basal diet + 0.3 mg·kg^−1^ sodium selenium + 0.3% H_2_O_2_ drinking water; (**F**) nano-selenium + H_2_O_2_ group: basal diet + 0.3 mg·kg^−1^ nano-selenium + 0.3% H_2_O_2_ drinking water.

**Figure 4:**
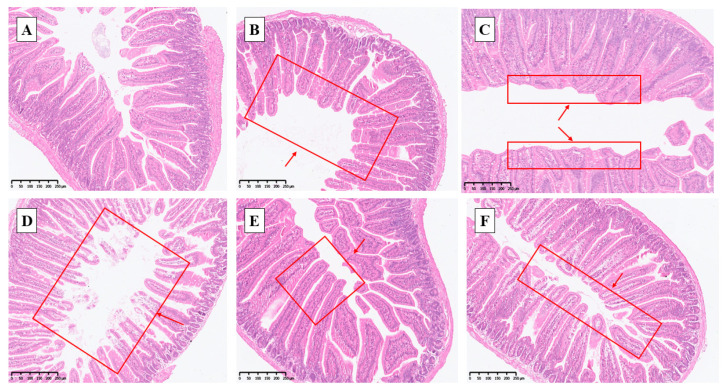
Diagram of jejunum sections in each group (HE staining, magnification: 100×). (**A**) Control: basal diet; (**B**) sodium selenite group: basal diet + 0.3 mg·kg^−1^ sodium selenium; (**C**) nano-selenium group: basal diet + 0.3 mg·kg^−1^ nano-selenium; (**D**) Control + H_2_O_2_ group: basal diet + 0.3% H_2_O_2_ drinking water; (**E**) sodium selenite + H_2_O_2_ group: basal diet + 0.3 mg·kg^−1^ sodium selenium + 0.3% H_2_O_2_ drinking water; (**F**) nano-selenium + H_2_O_2_ group: basal diet + 0.3 mg·kg^−1^ nano-selenium + 0.3% H_2_O_2_ drinking water.

Under normal conditions, the jejunal villi were arranged more neatly and completely after the addition of selenium (Figure 3B,C and Figure 4B,C; as shown in the red box indicated by the arrow), and nano-selenium was the best (Figure 3C and Figure 4C). After H_2_O_2_ stress, the jejunal villi of mice became disordered, incomplete, and almost incomplete, indicating that they were subjected to certain oxidative damage (Figure 3D and Figure 4D). This phenomenon was reversed in the sodium selenite group (Figure 3E and Figure 4E), while more complete and orderly villi were observed in the nano-selenium group (Figure 3F and Figure 4F). These results indicated that nano-selenium had a positive protective effect on the oxidative damage of villi induced by H_2_O_2_.

**Figure 5:**
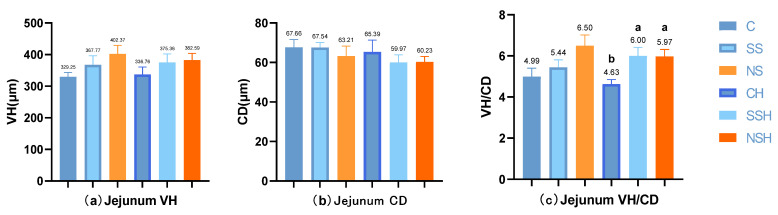

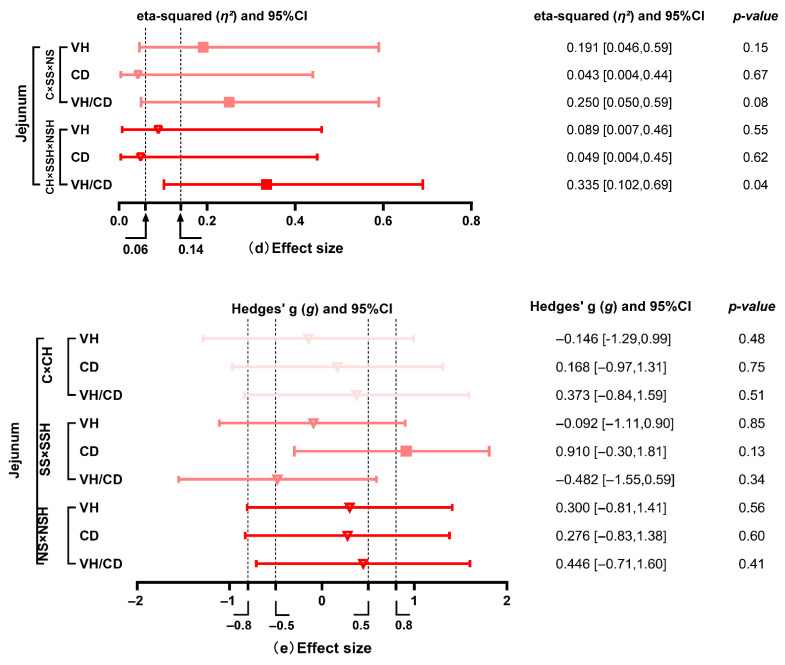
Effects of nano-selenium on jejunal tissue morphology under normal conditions or H_2_O_2_ oxidative stress treatment. (**a**) Bar graph of jejunal villus height index (VH), (**b**) bar graph of jejunal crypt depth index (CD), (**c**) bar graph of the ratio of VH to CD (VH/CD), (**d**) effect size forest plot of jejunum morphology [eta-squared (*η^2^*)], (**e**) effect size forest plot of jejunum morphology [Hedges’ g (*g*)]. Note: In all effect size forest plots, the squares and inverted triangles represent the effect size values. The squares indicate large effect sizes, and the lines on both sides represent their confidence intervals.

There were no significant differences in jejunum villus height and crypt depth among different selenium sources or between normal and H_2_O_2_-treated mice (*p* > 0.05, Figure 5). But the villus height has a relatively large effect size (*p* > 0.05, *η^2^* = 0.191, 95% CI [0.046,0.59]), nano-selenium is the most effective. Meanwhile, in the group SS and the group SSH, there was no significant difference between the two groups, but they had a relatively large effect size (*p* > 0.05, *g* = 0.910, 95% CI [−0.30,1.81]) for CD.

Under normal conditions, the group SS and group NS tended to increase the jejunal VH/CD one by one, and the effect size was large (*p* = 0.08, *η^2^* = 0.250, 95% CI [0.050,0.59]). However, in the presence of H_2_O_2_, the group SSH and group NSH significantly increased the VH/CD compared with the group CH, and the effect size was large (*p* < 0.05, *η^2^* = 0.335, 95% CI [0.102,0.69]).

### 3.3. Effect of Nano-Selenium on the Expression of Anti-Inflammatory and Antioxidant-Related Genes in the Intestine

#### 3.3.1. Gene Expression of Intestinal Inflammatory Factors

**Figure 6:**
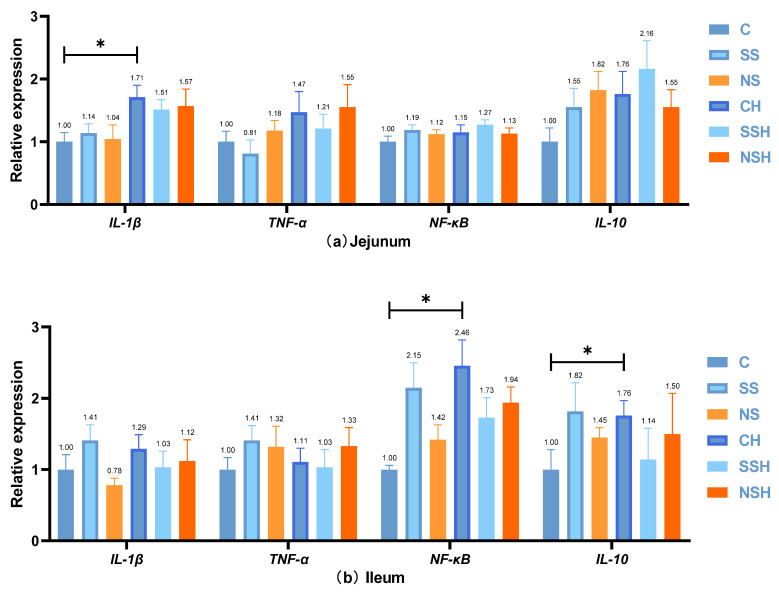

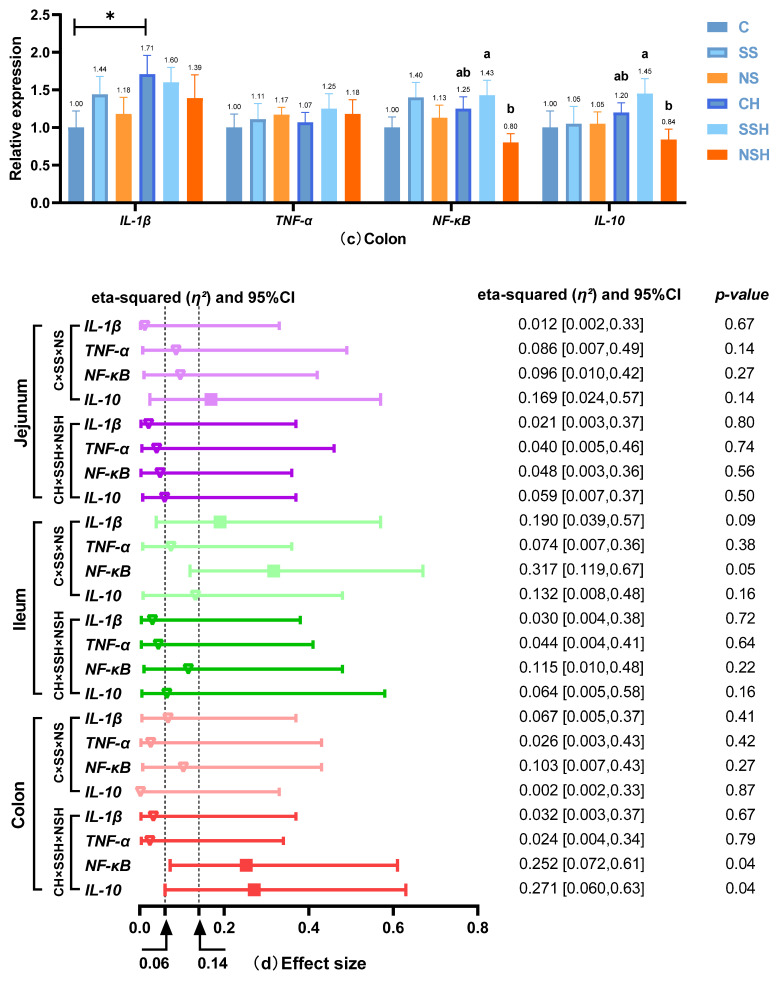

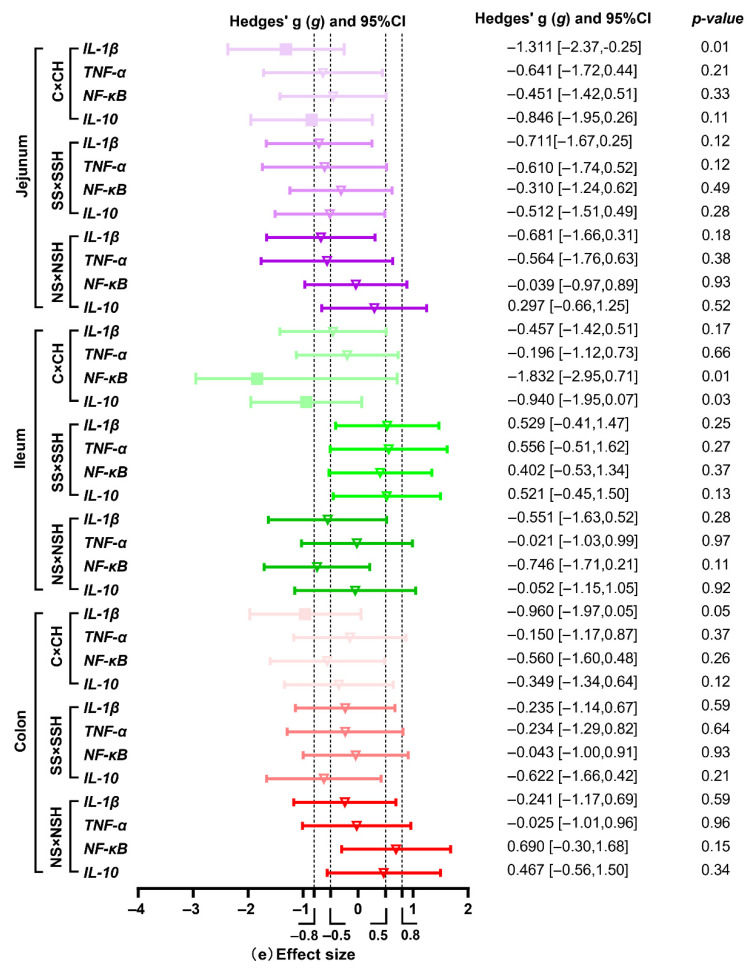
Effect of nano-selenium on gene expression levels of inflammatory factors in mouse gut under normal conditions or H_2_O_2_ oxidative stress treatment. (**a**) Bar graph of jejunal inflammatory factor gene expression, (**b**) bar graph of gene expression of inflammatory factors in the ileum, (**c**) bar graph of colonic inflammatory factor gene expression, (**d**) effect size forest plot of intestinal inflammatory factor gene expression [eta-squared (*η^2^*)], (**e**) effect size forest plot of intestinal inflammatory factor gene expression [Hedges’ g (*g*)].

In the jejunum of mice, sodium selenite and nano-selenium had no significant effect on the expression of inflammatory factors under normal conditions and H_2_O_2_ treatment (*p* > 0.05, Figure 6a,d,e). However, in normal conditions, the addition of selenium did not show a significant difference in *IL-10* levels, but the effect size was large (*p* > 0.05, *η^2^* = 0.169, 95% CI [0.024,0.57]). And in group C and CH, there was no significant difference between the two groups of *IL-10*, but the effect size was large (*p* > 0.05, *g* = −0.846, 95% CI [−1.95,0.26]). Compared with group C, group CH significantly increased the expression of *IL-1β*, and the effect size was large (*p* < 0.05, *g* = −1.311, 95% CI [−2.37,−0.25]).

In the ileum of mice, compared with group C, group SS tended to increase the expression of *IL-1β*, while group NS tended to decrease the expression of *IL-1β*, and the effect size was large (*p* = 0.09, *η^2^* = 0.190, 95% CI [0.039,0.57], Figure 6b,d,e). Compared with the group C and group NS, group SS tended to increase the expression of *NF-κB*, and the effect size was large (*p* = 0.05, *η^2^* = 0.317, 95% CI [0.119,0.67]). Compared with group C, group CH significantly increased the expression of *NF-κB* and *IL-10*, and the effect size was large (*p* < 0.05, |*g*| ≥ 0.8).

In the colon of mice, under normal conditions, sodium selenite and nano-selenium had no significant effect on the expression of colonic inflammatory factors in mice, and the effect size is small or medium (*p* > 0.05, *η^2^* < 0.14, |*g*| < 0.8, Figure 6c,d,e). Compared with group C, group CH significantly increased the expression of *IL-1β*, and the effect size was large (*p* < 0.05, *g* = −0.960, 95% CI [−1.97,0.05]). Compared with group SS, group NS significantly reduced the expression of *NF-κB* and *IL-10*, and the effect size was large (*p* < 0.05, *η^2^* ≥ 0.14).

#### 3.3.2. Intestinal Antioxidant Gene Expression

**Figure 7:**
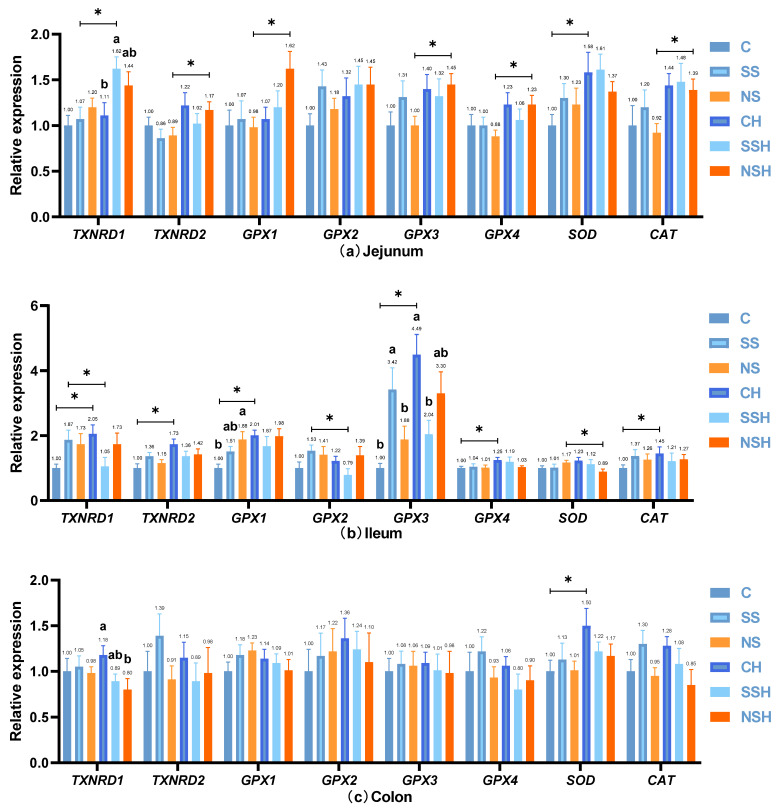

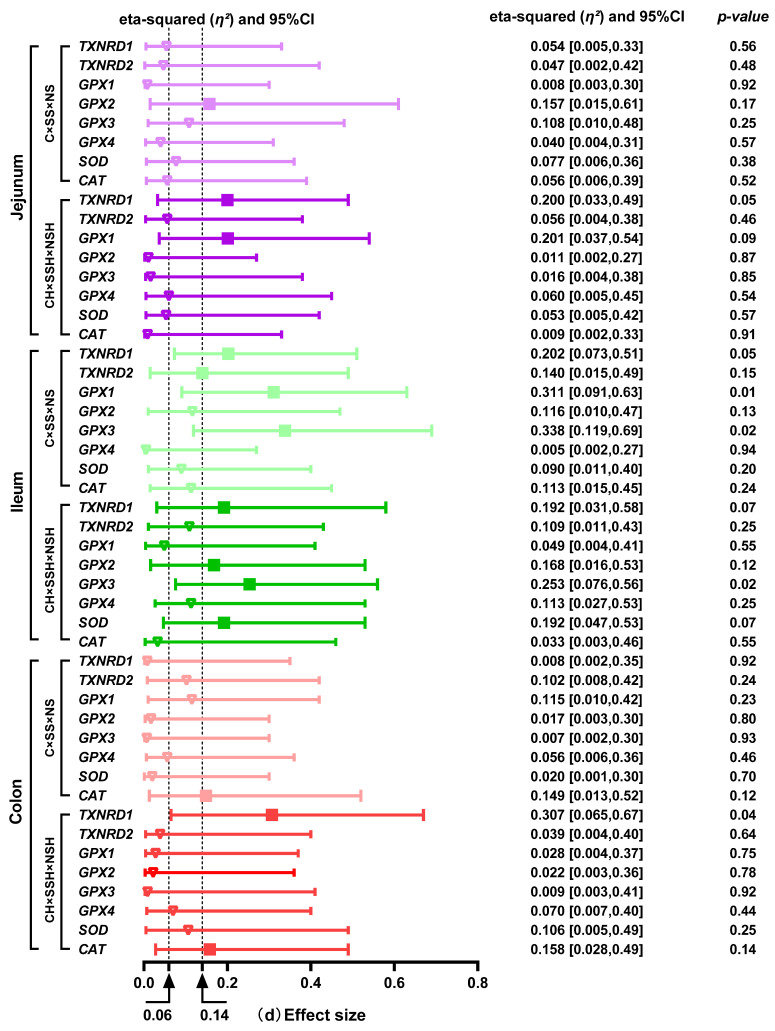

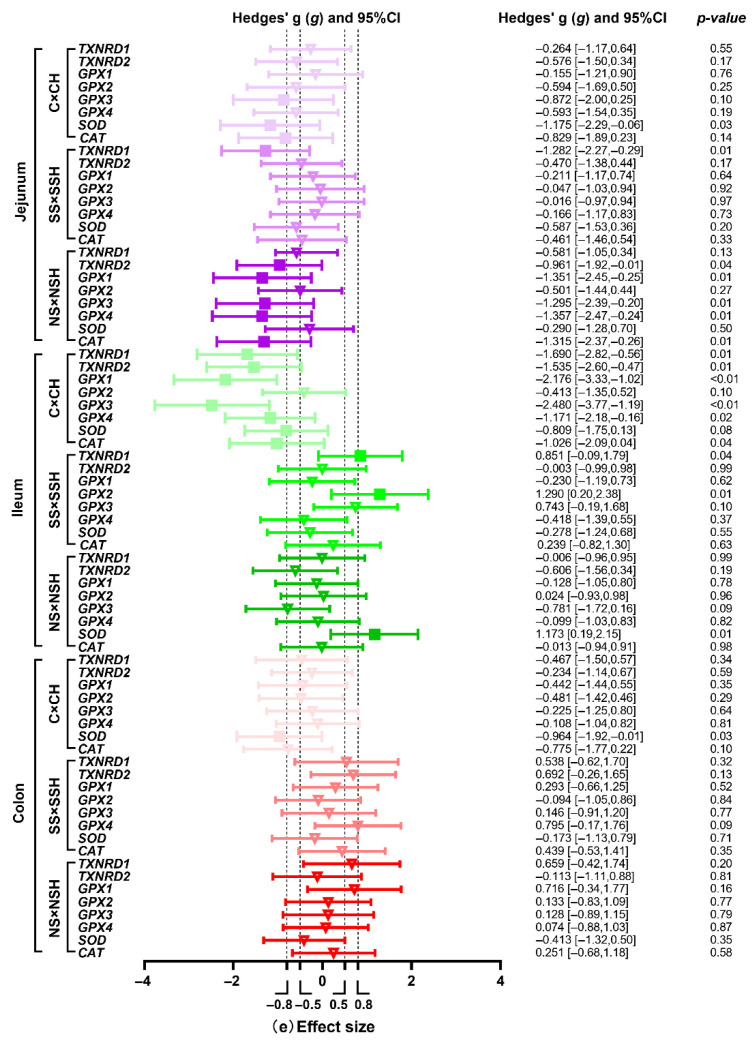
Effect of nano-selenium on gene expression levels of intestinal selenoprotein and antioxidant enzymes in mice under normal conditions or H_2_O_2_ oxidative stress treatment. (**a**) Bar graph of jejunal intestinal selenoprotein and antioxidant enzymes gene expression, (**b**) bar graph of gene expression of intestinal selenoprotein and antioxidant enzymes in the ileum, (**c**) bar graph of colonic intestinal selenoprotein and antioxidant enzymes gene expression, (**d**) effect size forest plot of intestinal selenoprotein and antioxidant enzymes gene expression [eta-squared (*η^2^*)], (**e**) effect size forest plot of intestinal selenoprotein and antioxidant enzymes gene expression [Hedges’ g (*g*)].

In the jejunum of mice, sodium selenite and nano-selenium had no significant effect on gene expression of antioxidant enzymes under normal conditions (*p* > 0.05, Figure 7a,d,e). However, the addition of selenium did not show a significant difference in *GPX2* levels, but the effect size was large (*p* > 0.05, *η^2^* = 0.157, 95% CI [0.015,0.61]). Compared with group C, group CH significantly increased the expression of *SOD* and the effect size was large (*p* < 0.05, *g* = −1.175, 95% CI [−2.29,−0.06]); meanwhile, there was no significant difference in *GPX3* and *CAT* between the two groups, but the effect size was large (*p* > 0.05, |*g*| ≥ 0.8). Compared with group SS, group SSH significantly increased the expression of *TXNRD1*, and the effect size was large (*p* < 0.05, *g* = −1.282, 95% CI [−2.27,−0.29]). Compared with group NS, group NSH significantly increased the expressions of *TXNRD2*, *GPX1*, *GPX3*, *GPX4*, and *CAT*, and the effect size was large (*p* < 0.05, |*g*| ≥ 0.8). In the presence of H_2_O_2,_ group SSH significantly increased the expression of *TXNRD1* and the effect size was large (*p* = 0.05, *η^2^* = 0.200, 95% CI [0.033,0.49]), and group NSH tended to increase the expression of *GPX1* and the effect size was large (*p* = 0.09, *η^2^* = 0.201, 95% CI [0.037,0.54]).

In the ileum of mice, under normal conditions, sodium selenite and nano-selenium had a tendency to increase the expression level of *TXNRD1* but the effect size was large (*p* = 0.05, *η^2^* = 0.202, 95% CI [0.073,0.51], Figure 7b,d,e), group NS significantly increased the expression level of *GPX1*, and the effect size was large compared with the group C (*p* < 0.05, *η^2^* = 0.311, 95% CI [0.091,0.63]). Compared with group C and group NS, group SS significantly increased *GPX3* expression level, and the effect size was large (*p* < 0.05, *η^2^* = 0.338, 95% CI [0.119,0.69]). However, the addition of selenium did not show a significant difference in *TXNRD2* levels, but the effect size was large (*p* < 0.05, *η^2^* = 0.140, 95% CI [0.015,0.49]). Compared with group C, group CH significantly increased the expression levels of *TXNRD1*, *TXNRD2*, *GPX1*, *GPX3*, *GPX4*, and *CAT*, and the effect size was large (*p* < 0.05, |*g*| ≥ 0.8), and tended to increase the expression level of *SOD* but the effect size was large (*p* = 0.08, *g* = −0.809, 95% CI [−1.75,0.13]). Compared with group SS, group SSH significantly reduced the expression of *TXNRD1* and *GPX2*, and the effect size was large (*p* < 0.05, |*g*| ≥ 0.8). Compared with group NS, group NSH tended to increase the expression of *GPX3* (*p* = 0.09), and significantly decreased the expression of *SOD*, and the effect size was large (*p* < 0.05, *g* = 1.173, 95% CI [0.19,2.15]). Compared with the group CH, group SSH tended to decrease *TXNRD1* and the effect size was large (*p* = 0.07, *η^2^* = 0.192, 95% CI [0.031,0.58]), and significantly decreased *GPX3* expression level and the effect size was large (*p* < 0.05, *η^2^* = 0.253, 95% CI [0.076,0.56]), and group NSH had a tendency to decrease *SOD* expression level and the effect size was large (*p* = 0.07, *η^2^* = 0.192, 95% CI [0.047,053]). However, in H_2_O_2_ treatment, the addition of selenium did not show a significant difference in *GPX2* level, but the effect size was large (*p* > 0.05, *η^2^* = 0.168, 95% CI [0.016,0.53]).

In the colon of mice, under normal conditions, sodium selenite and nano-selenium had no significant effect on the expression of antioxidant enzymes (*p* > 0.05, Figure 7c,d,e). However, the addition of selenium to the *CAT* did not show significant effects, but the effect size was large (*p* > 0.05, *η^2^* = 0.149, 95% CI [0.013,0.52]). Compared with group C, group CH significantly increased the expression of *SOD*, and the effect size was large (*p* < 0.05, *g* = −0.964, 95% CI [−1.92,−0.01]). Compared with group SS, group SSH tended to reduce the expression of *GPX4* (*p* = 0.09). In the H_2_O_2_ treatment, nano-selenium significantly reduced the expression of TXNRD1, with a large effect size (*p* < 0.05, *η*^2^ = 0.307, 95% CI [0.065, 0.67]). However, the addition of selenium did not show a significant difference in *CAT* levels, but the effect size was large (*p* > 0.05, *η^2^* = 0.158, 95% CI [0.028,0.49]).

### 3.4. Effect of Nano-Selenium on the Antioxidant Capacity

In the jejunum of mice, group SS tended to increase T-SOD, and the effect size was large (*p* = 0.07, *η^2^* = 0.181, 95% CI [0.026,0.47], Table 4). Compared with group C and group SS, group NS significantly reduced T-AOC (*p* < 0.05, *η^2^* = 0.308, 95% CI [0.087,0.61]). However, in normal conditions, the addition of selenium did not show a significant difference in MDA levels, but the effect size was large (*p* > 0.05, *η^2^* = 0.168, 95% CI [0.019,0.50]), nano-selenium effectively reduced the content of MDA, but the sodium selenite group had a certain increase. Compared with group C, group CH significantly reduced T-AOC, and the effect size was large (*p* < 0.05, *g* = 1.857, 95% CI [0.77,2.95]). Compared with the group SS, group SSH tended to reduce T-AOC (*p* = 0.06). Compared with group SSH, group NSH significantly reduced CAT, and the effect size was large (*p* < 0.05, *η^2^* = 0.249, 95% CI [0.047,0.60]).

In the ileum of mice, sodium selenite and nano-selenium had no significant effect on the antioxidant capacity under normal conditions and H_2_O_2_ treatment (*p* > 0.05). Compared with the group SS, group SSH significantly increased MDA, and the effect size was large (*p* < 0.05, *g* = −1.600, 95% CI [−2.84,−0.36]). Compared with the group NS, group NSH tended to increase MDA, and the effect size was large (*p* = 0.06, *g* = −1.103, 95% CI [−2.18,−0.03]).

In the colon of mice, compared with group C, group SS significantly increased T-SOD, and the effect size was large (*p* < 0.05, *η^2^* = 0.285, 95% CI [0.064,0.66]). Compared with group SS, group SSH significantly reduced T-SOD and T-AOC, and the effect size was large (*p* < 0.05, |*g*| ≥ 0.8). Compared with group NS, group NSH significantly reduced T-SOD, and the effect size was large (*p* < 0.05, *g* = 0.967, 95% CI [0.01,1.92]). Compared with group CH, group SSH significantly reduced T-SOD, and the effect size was large (*p* < 0.05, *η^2^* = 0.242, 95% CI [0.081,0.54]). Group SSH significantly reduced T-AOC compared with the group CH and group NSH, and the effect size was large (*p* < 0.05, *η^2^* = 0.323, 95% CI [0.122,0.63]).

## 4. Discussion

### 4.1. The Role of Chitosan in Nano-Selenium Materials

The nano-selenium material used in the present study was synthesized using chitosan powder, so its components include selenium and chitosan. At present, chitosan has been widely applied in the field of nanomaterials [16]. Chitosan nano-selenium particles (CSNP), as nano-carriers, have good stability because they have a high positive charge, which can prevent the aggregation and sedimentation of nanoparticles; CSNP has a large specific surface area, which enables it to have a high loading capacity and encapsulation for substances. Meanwhile, CSNP has a high degree of biocompatibility, good tolerance to organisms, and does not cause obvious adverse reactions [17]. Therefore, chitosan has excellent capabilities in terms of solubility, biocompatibility, biodegradability, stability, and easy functionalization. Moreover, it has the advantages of being non-toxic, having a low-level immune response, mucosal adhesion, and high absorption [18,19]. However, due to its relatively weak mechanical properties and high degradation rate, it is usually used in combination with other substances or in composite materials [20]. So, from a biological activity point of view, chitosan mainly serves as a carrier in the form of nanoparticles to effectively transport and deliver substances in organisms, such as drugs in medicine and trace elements in nutrition.

### 4.2. Effect of Nano-Selenium on Organ Index

The organ index (organ-to-body weight ratio) is an important indicator of physiological status and toxicity in animals. Under normal conditions, organ indices remain stable; significant increases may indicate edema or hyperplasia, while decreases suggest atrophy [21,22,23,24]. In this study, there were no significant differences in the organ indices of liver, kidney, heart, and pancreas of mice among different treatments, indicating that the organ indices of mice were stable against selenium supplementation and H_2_O_2_ treatment, and were not affected by selenium or the toxic effects of H_2_O_2_. Although not statistically significant, a large effect size suggested potential influences of selenium and H_2_O_2_ on the kidney index. However, both sodium selenite and nano-selenium significantly reduced the spleen index in mice. Similar to the study by Attia et al. [25], selenium supplementation significantly reduced the organ index in the spleen of chickens, while other organ indices were not significantly affected by selenium level or selenium source. In the study results of Ali et al. [26], the spleen index was also reduced when chickens were given selenium. On the one hand, it may be related to the experimental time, experimental conditions, and individual animal differences. On the other hand, selenium may be present in the basal diet of mice, and additional supplementation of selenium may have an effect on their spleen. When nano-selenium was fed in the present study, H_2_O_2_ treatment significantly increased the spleen index of mice. As mentioned above, H_2_O_2_ may have caused edema or lesions in the spleen of mice, resulting in an increase in spleen index.

### 4.3. Effect of Nano-Selenium on the Morphology of Jejunum

The main function of the jejunum is to digest and absorb nutrients, which contributes to the normal operation of the body’s metabolism and immune system [27]. The villus height (VH), crypt depth (CD), and the ratio of villus height to crypt depth (VH/CD) are key indicators of intestinal health and absorptive capacity [28,29]. Higher villus height indicates greater intestinal absorption area and better absorption ability [30]. Crypt depth was closely related to the proliferation rate of epithelial cells [31]. Shallow crypts suggest higher cell maturation, whereas deeper crypts indicate villus atrophy and reduced function [32]. The ratio of villus height to crypt depth (VH/CD) is a comprehensive reflection of intestinal function.

In this study, sodium selenite and nano-selenium had no significant effect on villus height and crypt depth in the jejunum of mice under normal conditions or H_2_O_2_ treatment. Similar to the results of this study, both sodium selenite and nano-selenium had no significant effect on jejunal villus height and crypt depth of broilers at 21 days. In addition, nano-selenium and sodium selenite also had no effect on jejunal villus height under stress [33]. Khajeh et al. [34] showed that nano-selenium had different effects on crypt depth at the height of jejunal villi in broilers at different days of age. Therefore, we speculate that this may be related to rearing duration. However, through the analysis of effect size, it was found that adding selenium had a large effect on the villus height, and the nano-selenium group had the highest villus height. This indicates that nano-selenium has certain effectiveness in improving the villus height in the jejunum of mice, but further proof is needed. We used H_2_O_2_ to induce oxidative stress, which had no effect on jejunum morphology in mice. Similar to the results of some studies, stress had no significant effect on the intestinal morphology of animals [33,35]. On the one hand, it may be related to the rapid epithelialization of intestinal mucosa [35]; on the other hand, when subjected to stress, the body will produce a self-protection mechanism to reduce the damage caused by stress. We found that supplementation of selenium under normal conditions tended to improve the jejunal VH/CD of mice, and the effect of nano-selenium was better. In the presence of H_2_O_2_, feeding sodium selenite and nano-selenium significantly increased the jejunal VH/CD. And the effect sizes are all considered to be large. Rehman et al. [33] also found that the addition of sodium selenite and nano-selenium significantly improved the VH/CD of the jejunum in broilers [34]. According to the comprehensive index of VH/CD, nano-selenium has a positive protective effect on jejunum morphology in mice.

From the analysis of results, selenium supplementation had no significant effect on villus height and crypt depth, but it had a certain effectiveness in improving the villus height, and significantly increased VH/CD. At the same time, from the numerical value and morphological diagram, H_2_O_2_ has a certain damage effect on the jejunum, and selenium supplementation can improve this phenomenon, and nano-selenium has a better effect. He et al. [36] pointed out that selenium deficiency in animals affects the structure of small intestinal mucosa and damages the functions of the small intestinal immune barrier and physical barrier. At the same time, selenium can also enhance the intestinal barrier by improving the intestinal immune status, oxidation status, and maintaining the integrity and normal function of the small intestine [37,38,39]. Therefore, nano-selenium has a positive effect on jejunum morphology in mice under stress.

### 4.4. Effect of Nano-Selenium on the Expression of Intestinal Inflammatory Factors

Intestinal immune cytokines are crucial in regulating gastrointestinal inflammation [40]. Key pro-inflammatory factors include *IL-1β*, which promotes neutrophil infiltration and T-cell activation [41], and *NF-κB*, a transcription factor that enhances multiple pro-inflammatory cytokines [42]. Conversely, the anti-inflammatory cytokine *IL-10*—produced by Th2 cells—alleviates inflammation by inhibiting pro-inflammatory cytokine production and plays a protective role in colitis models [41,43,44,45]. Reduced *IL-10* levels weaken anti-inflammatory capacity and exacerbate inflammation. Inflammatory pathways are often redox-sensitive; excess ROS induces oxidative stress, promoting inflammation and tissue apoptosis [46].

We measured inflammatory factor expression across intestinal segments in mice. Under normal conditions, selenium supplementation did not significantly alter expression levels; the expression of intestinal inflammatory factors was at normal levels and did not seem to be regulated by selenium. Nonetheless, large effect sizes were observed for certain indicators: nano-selenium increased *IL-10* expression in the jejunum, while in the ileum, it reduced *IL-1β*—unlike sodium selenite, which elevated both *IL-1β* and *NF-κB*. These trends suggest a potential anti-inflammatory effect of nano-selenium that merits further study. H_2_O_2_ treatment up-regulates several inflammatory factors in all three intestinal segments. Similar to previous studies, H_2_O_2_ up-regulates the expression of some inflammatory factors in intestinal epithelial cells [47,48,49]. As an ROS, H_2_O_2_ disrupts redox balance, promotes oxidative stress, and contributes to intestinal diseases [50]. In this study, H_2_O_2_ up-regulated the expression of pro-inflammatory factors in different intestinal segments, which suggests that feeding H_2_O_2_ in drinking water induces an inflammatory response in the intestine of mice to a certain extent, and the intestine of mice is under stress. In the jejunum, although not significantly, H_2_O_2_ had a large effect size on increasing the expression of *IL-10*. In the ileum, H_2_O_2_ up-regulated the expression of anti-inflammatory factor *IL-10*, which may be a self-protective mechanism of the body to secrete anti-inflammatory cytokines by up-regulating the expression of anti-inflammatory factors, thereby slowing down the occurrence of inflammation. Through longitudinal comparison, we found that H_2_O_2_ had no significant effect on the expression of *IL-1β*, *NF-κB*, and *IL-10* in the treatment of selenium supplementation. Selenium supplementation appeared to normalize the expression of these inflammatory factors. At the same time, the expression levels of *NF-κB* and *IL-10* in the colon of mice in the nano-selenium group were the lowest in the presence of H_2_O_2_. From a signaling pathway perspective, the *NF-κB* pathway plays a key role in mediating immune and inflammatory responses. As a major regulator of inflammatory responses, its role is mainly to regulate the expression of hundreds of immune-related genes, especially those encoding pro-inflammatory cytokines and chemokines [51]. The *NF-κB* pathway mediates inflammatory responses via classical (e.g., TLR, TNFR) and non-classical (e.g., CD40, RANK) activation [52]. Its complex interaction with ROS may involve both activation and inhibition. This difference may be related to the specificity of different upstream pathways and cells [53]. Studies have found that ROS drives the *NF-κB* pathway in the duodenum of mice, thereby inducing inflammation [54]. Therefore, on the one hand, ROS directly regulates the *NF-κB* signaling pathway; on the other hand, some cytokines (such as *TNF-α* and *IL-1β*) activate *NF-κB*, and the activated *NF-κB* in turn drives the expression of pro-inflammatory factors to participate in the inflammatory process [52,55]. In this study, H_2_O_2_ increased *IL-1β* (jejunum/colon). In the ileum, *NF-κB* was also significantly elevated, accompanied by a numerical increase in *IL-1β* levels. These observations suggest that H_2_O_2_, as a reactive oxygen species (ROS), exerts pro-inflammatory effects through dual mechanisms: (1) up-regulating cytokine *IL-1β* to activate the *NF-κB* signaling pathway, and (2) directly stimulating *NF-κB* pathway activation. However, in the three groups fed with H_2_O_2_ drinking water, compared with sodium selenite, nano-selenium down-regulated the expression of *NF-κB*, and compared with the Control group fed with H_2_O_2_ drinking water, *IL-1β* and *NF-κB* were decreased numerically, indicating anti-inflammatory potential. This finding aligns with numerous studies demonstrating selenium’s ability to mitigate intestinal stress-induced inflammation through modulation of inflammatory mediators [56,57,58,59,60,61]. For example, some studies have found that nano-selenium significantly down-regulate the expression levels of *IL-1β* and *TNF-α* [62,63].

Selenium exerts pivotal regulatory functions in intestinal inflammation. Accumulating evidence demonstrates that selenium deficiency in animals triggers oxidative stress, up-regulates the expression of corresponding inflammatory factors, and induces intestinal tissue inflammation [64,65,66]. This deficiency is also associated with reduced antimicrobial peptide production [67] and disrupted gut microbiota equilibrium [68]. Selenium exerts antioxidant and immunomodulatory effects via selenoproteins [69]. Notably, nano-selenium demonstrates enhanced bioavailability and biosafety profiles attributable to its unique nanostructure, thereby exhibiting modulatory effects on intestinal inflammatory markers.

### 4.5. Effect of Nano-Selenium on the Expression of Intestinal Selenoproteins and Antioxidant Enzymes

Selenium mainly exists in the form of selenocysteine (SeCys) in the body [70], and then selenocysteine is inserted into the nascent polypeptide chain in a co-translational manner by insertion elements, thereby synthesizing selenoproteins [71]. A total of 25 selenoprotein genes were identified by bioinformatics studies [72]. As antioxidant enzymes, selenoproteins such as glutathione peroxidase (*GPX*) family, such as *GPX1-4*, can effectively metabolize peroxides in cells and act as an antioxidant defense mechanism to prevent excessive reactive oxygen species/reactive nitrogen species. Thioredoxin reductase (*TXNRD1-3*) are oxidoreductases that regulate the redox state of proteins such as thioredoxin as well as small molecules such as thioctic tetrathionate [73]. These, along with enzymes like superoxide dismutase (*SOD*) and catalase (*CAT*), are essential in mitigating oxidative stress.

This study investigated the transcriptional regulation of antioxidant markers (selenoproteins and antioxidases). Under normal conditions, neither sodium selenite nor nano-selenium supplementation significantly modulated the expression of most antioxidant markers in the mouse intestine, consistent with previous findings demonstrating no alteration in *SOD* and *CAT* gene expression upon selenium supplementation [74]. We speculate that the regulation of antioxidant marker expression may be related to the dose level of selenium supplementation and the physiological status of the animals. Several studies have found that the regulation of selenoprotein expression may require a supertrophic dose of selenium [75,76]. Notably, selenium supplementation has been demonstrated to enhance the body’s antioxidant defense system predominantly under oxidative stress conditions [77,78], potentially explaining the lack of significant effects observed under basal conditions. Although not statistically significant, large effect sizes were observed for certain indicators: selenium increased *GPX2* in the jejunum, *TXNRD1* and *TXNRD2* in the ileum, and *CAT* in the colon. Only in the ileum did nano-selenium significantly up-regulate *GPX1*, and sodium selenite increased *GPX3*, aligning with reports that selenium can up-regulate *GPX* family expression [79,80,81]. Selenoproteins have a unique and graded dependence on the trace element selenium [82]. H_2_O_2_ treatment in mice fed nano-selenium significantly increased the expression of antioxidant markers and concentrated in the jejunum. Labunskyy et al. pointed out that some selenoproteins are called stress-related selenoproteins [71], and these proteins are closely related to the stress state in the body [83]. Selenium supplementation was found to up-regulate selenoprotein expression under stress conditions [84,85,86]. These results indicate that selenium plays a positive role in the expression of selenoproteins and antioxidants in the body under stress. Due to its small volume, large specific surface area, and unique physicochemical properties, nano-selenium has higher antioxidant activity and better bioavailability [87,88], thus significantly improving the antioxidant status of mice intestinal tract. However, in the basal diet, H_2_O_2_ also up-regulated the expression of some antioxidant markers, mainly concentrated in the ileum of mice. From another point of view, we speculate that this is a part of the compensatory response to prevent oxidative damage, which is a kind of self-protection reaction of the body [3]. Te Velde et al. also pointed out that up-regulation of selenoproteins or antioxidant enzymes may be a defense mechanism against oxidative stress during inflammation [89]. Similarly, some studies have found that the expression of selenoproteins and antioxidant enzymes is up-regulated under stress, and the expression of selenoproteins and antioxidant enzymes is restored to normal level with the addition of selenium, such as nano-selenium [57,90,91]. So, in the ileum, H_2_O_2_ treatment had no effect on most of the antioxidant markers in mice fed nano-selenium.

Overall, there were differences in the expression of antioxidant markers in different intestinal segments of mice. The expression of selenoproteins is affected by many factors, and the different expression may be related to the status of selenium, the catabolic pathways of selenium in different tissues, and the differences in its biosynthesis and utilization [69]. It is also regulated by differences in the availability of selenium [71]. In addition, intestinal microorganisms can also affect the expression of selenoproteins. Guevara et al. [81] pointed out that selenium affects the composition of microbes in different intestinal segments. Different microorganisms have different uptake, storage, utilization, and excretion abilities of selenium, which may lead to competition between microorganisms and the host for selenium, limiting the availability of selenium and affecting the expression of selenoproteins [92,93].

### 4.6. Effect of Nano-Selenium on Intestinal Antioxidation

T-SOD, T-AOC, CAT, and MDA are the main indices reflecting the antioxidant capacity and redox status of the body. T-SOD is the first line of antioxidant defense, which can convert superoxide anions into H_2_O_2_ and water [94]. T-AOC reflects the overall levels of enzymatic and non-enzymatic antioxidants in the body [95]. CAT, as its name implies, is an enzyme that decomposes H_2_O_2_. H_2_O_2_ produced by the body and superoxide can be degraded to water and oxygen by CAT [96]. One molecule of CAT can convert 6 million H_2_O_2_ grabs into water and oxygen per minute [97], thus playing a key role in antioxidant protection. Conversely, MDA—a product of lipid peroxidation induced by ROS—serves as a biomarker of oxidative stress and cellular membrane damage [98,99].

This study revealed that nano-selenium supplementation reduced the T-AOC of the jejunum under normal conditions, which may be related to the toxic effects of nano-selenium. Long-term study by Xiao et al. [100] demonstrated that nano-selenium can suppress both antioxidant enzyme activity and selenoprotein expression, thereby exacerbating oxidative stress, which leads to organ lesions and decreased antioxidant capacity. The toxicity of nano-selenium is critically dependent on the size and chemical properties of nanoparticles [101]. In addition, both sodium selenite and nano-selenium have pro-oxidative properties at high concentrations, leading to the generation of ROS [102]. Therefore, we speculate that the jejunum’s anatomical position as the primary intestinal segment exposed to nano-selenium first, and may suffer from toxic effects to a certain extent, so the T-AOC is reduced; however, this might also be an accidental phenomenon. Although not statistically significant, sodium selenite increased MDA content in the jejunum, whereas nano-selenium reduced it, with a large effect size, warranting further investigation. Meanwhile, sodium selenite seems to be more toxic than nano-selenium, which was demonstrated in a short-term toxicity test [103]. It up-regulated ileal IL-1β and NF-κB expression, increased jejunal MDA, and reduced colonic T-SOD and T-AOC in H_2_O_2_-treated mice. Notably, sodium selenite increased colonic T-SOD under normal conditions but decreased it under oxidative stress, suggesting it may aggravate H_2_O_2_-induced damage.

Collectively, these findings indicate that selenium supplementation—particularly sodium selenite—may exert adverse effects on select intestinal antioxidant parameters, though the overall impact on systemic antioxidant capacity appears limited. Previous studies also reported no significant effects of organic selenium or nano-selenium glycinate on T-SOD, MDA, or T-AOC [104,105]. We propose that selenium supplementation effects may be modulated by three key factors: (1) treatment duration, (2) dosage levels, and (3) selenium bioavailability. Furthermore, as previously discussed, gut microbial selenium competition may represent an additional limiting factor for host selenium utilization, though this hypothesis requires experimental validation.

### 4.7. Limitations of This Study and Future Research Directions

This study has several limitations. Primarily, although the nano-selenium was synthesized using chitosan, no chitosan-only Control group was included. Therefore, the potential influence of chitosan itself on bioavailability and bioactivity remains unassessed. Furthermore, certain negative effects were observed in intestinal antioxidant indicators following selenium supplementation. Although we hypothesize that these may be related to selenium toxicity, this study did not include explicit toxicity assays or dose–response evaluations, which warrants further investigation.

In terms of statistical analysis, although all statistically significant results showed large effect sizes (*p* < 0.05, *η^2^* ≥ 0.14, |*g*| ≥ 0.8), several non-significant outcomes also exhibited large effect sizes. This indicates that it may have certain practical significance and some effect, but there is no significant evidence to support this effect. The wide confidence intervals of some effect estimates further reflect uncertainty in the true effect magnitude, likely resulting from the small sample size. Finally, due to limited tissue availability, only gene expression of selected inflammatory and antioxidant markers was measured; protein-level validation was not performed.

Based on these limitations, subsequent investigations should focus on the following aspects:

1. Include a chitosan-only experimental group to clarify its individual contribution to the effects of nano-selenium.

2. Perform toxicity and dose–response studies using graded concentrations of nano-selenium, combined with assays such as LDH and MTT, to establish its safety profile.

3. Increase sample size to enhance statistical power, reduce random error, and obtain more precise effect estimates.

4. Supplement gene expression data with Western blot analysis to corroborate findings at the protein level.

5. Conduct mechanistic studies using cell models to elucidate underlying signaling pathways.

## 5. Conclusions

Our results showed that 0.3% H_2_O_2_ drinking water induced intestinal oxidative stress in mice to a certain extent. The supplementation with sodium selenium and nano-selenium improved intestinal morphology, alleviated oxidative damage, and restored the expression of related inflammatory factors. At the same time, nano-selenium played a positive role in increasing the expression of selenoprotein in the jejunum under stress. However, it showed no significant effect on improving the antioxidant indicators in the intestinal tract. In the future, further research is needed from the perspectives of carriers, selenium dosage levels, protein levels, and increasing the sample size.

## Figures and Tables

**Table 1:** Ingredient composition of basal diets (as-fed basis).

Composition	Ingredient (g·kg^−1^)
Casein	200
Maltodextrin	100
Corn starch	430.1
Sucrose	80
Fat powder 1-extruded corn type	100
Microcrystalline cellulose	50
L-cystine	3
Calcium carbonate	6
Calcium hydrogen phosphate	10
Potassium citrate	8
Choline chloride, 50%	2.6
Vitamin ^1^	0.3
Mineral element ^2^	10
Total	1000

^1^ Per kilogram diet provided: vitamin A, 7200 IU; vitamin D_3_, 1440 IU; vitamin E, 60 IU; vitamin K_3_, 2.88 mg; vitamin B_1_, 1.2 mg; vitamin B_2_, 4.32 mg; vitamin B_6_, 1.26 mg; vitamin B_12_, 0.15 mg; D-biotin, 2.88 mg; D-pantothenic acid, 15 mg; folate, 2.4 mg; niacin, 24 mg. ^2^ Per kilogram diet provided: sodium, 1.022 g; calcium, 6.2626 g; phosphorus, 2.9932 g; chloride, 1.8119 g; zinc, 0.0273 g; copper, 0.0053 g; iodine, 0.005 g; potassium, 3.06 g; manganese, 0.065 g; iron, 0.0567 g; magnesium, 0.4781 g.

**Table 2:** Primer sequence.

Gene Name	Sequence	Base Pair	Accession No.
*β-actin*	F: GGCTGTATTCCCCTCCATCG R: CCAGTTGGTAACAATGCCATGT	154	NM_007393
*IL-1β*	F: GCAACTGTTCCTGAACTCAACT R: ATCTTTTGGGGTCCGTCAACT	89	NM_008361
*TNF-α*	F: CCCTCACACTCAGATCATCTTCT R: GCTACGACGTGGGCTACAG	61	NM_013693
*NF-κB*	F: ATGGCAGACGATGATCCCTAC R: TGTTGACAGTGGTATTTCTGGTG	111	NM_008689
*IL-10*	F: GCTCTTACTGACTGGCATGAG R: CGCAGCTCTAGGAGCATGTG	105	NM_010548
*TXNRD1*	F: CCCACTTGCCCCAACTGTT R: GGGAGTGTCTTGGAGGGAC	134	NM_001042523
*TXNRD2*	F: GATCCGGTGGCCTAGCTTG R: TCGGGGAGAAGGTTCCACAT	86	NM_013711
*GPX1*	F: AGTCCACCGTGTATGCCTTCT R: GAGACGCGACATTCTCAATGA	105	NM_008160
*GPX2*	F: GCCTCAAGTATGTCCGACCTG R: GGAGAACGGGTCATCATAAGGG	143	NM_030677
*GPX3*	F: CCTTTTAAGCAGTATGCAGGCA R: CAAGCCAAATGGCCCAAGTT	120	NM_008161
*GPX4*	F: GATGGAGCCCATTCCTGAACC R: CCCTGTACTTATCCAGGCAGA	185	NM_008162
*SOD*	F: CAGACCTGCCTTACGACTATGG R: CTCGGTGGCGTTGAGATTGTT	113	NM_013671
*CAT*	F: AGCGACCAGATGAAGCAGTG R: TCCGCTCTCTGTCAAAGTGTG	181	NM_009804

*β-actin*: beta-actin; *IL-1β*: interleukin-1β; *TNF-α*: tumor necrosis factor-*α*; *NF-κB*: nuclear factor kappa B; *IL-10*: interleukin-10; *TXNRD1*: thioredoxin reductase 1; *TXNRD2*: thioredoxin reductase 2; *GPX1*: glutathione peroxidase 1; *GPX2*: glutathione peroxidase 2; *GPX3*: glutathione peroxidase 3; *GPX4*: glutathione peroxidase 4; *SOD*: superoxide dismutase; *CAT*: catalase.

**Table 3:** Effects of nano-selenium on organ index under normal conditions or H_2_O_2_ oxidative stress treatment.

Index	Treatments										
	C	SS		NS		CH		SSH		NSH	
Liver	0.044 ± 0.001	0.042 ± 0.002		0.044 ± 0.001		0.044 ± 0.001		0.042 ± 0.001		0.044 ± 0.001	
Spleen	0.0043 ± 0.0003 ^a^	0.0035 ± 0.0002 ^b^		0.0036 ± 0.0002 ^b^		0.0042 ± 0.0002		0.0038 ± 0.0003		0.0042 ± 0.0002 *	
Kidney	0.0066 ± 0.0002	0.0064 ± 0.0003		0.0071 ± 0.0002		0.0070 ± 0.0002		0.0067 ± 0.0002		0.0065 ± 0.0002	
Heart	0.0050 ± 0.0001	0.0048 ± 0.0002		0.0050 ± 0.0002		0.0048 ± 0.0002		0.0047 ± 0.0002		0.0048 ± 0.0001	
Pancreas	0.0050 ± 0.0002	0.0048 ± 0.0002		0.0053 ± 0.0002		0.0049 ± 0.0002		0.0053 ± 0.0002		0.0049 ± 0.0003	
		**C × SS × NS**		**CH × SSH × NSH**		**C × CH**		**SS × SSH**		**NS × NSH**	
		*p*-value	*η^2^*-value (95% CI)	*p*-value	*η^2^*-value (95% CI)	*p*-value	*g*-value (95% CI)	*p*-value	*g*-value (95% CI)	*p*-value	*g*-value (95% CI)
Liver		0.33	0.083 [0.004,0.44]	0.67	0.031 [0.004,0.34]	0.71	0.161 [−0.74,1.06]	0.85	−0.082 [−1.01,0.84]	0.97	−0.016 [−0.94,0.91]
Spleen		0.04	0.219 [0.036,0.52]	0.38	0.070 [0.003,0.40]	0.80	0.111 [−0.79,1.01]	0.44	−0.340 [−1.25,0.57]	0.03	−1.011 [−2.00,−0.02]
Kidney		0.12	0.147 [0.016,0.50]	0.22	0.109 [0.011,0.44]	0.23	−0.533 [−1.45,0.38]	0.33	−0.443 [−1.38,0.50]	0.05	0.891 [−0.06,1.84]
Heart		0.76	0.021 [0.003,0.31]	0.76	0.021 [0.002,0.34]	0.62	0.223 [−0.71,1.15]	0.52	0.283 [−0.62,1.19]	0.27	0.497 [−0.44,1.44]
Pancreas		0.31	0.085 [0.008,0.37]	0.47	0.055 [0.004,0.32]	0.73	0.151 [−0.75,1.05]	0.13	−0.702 [−1.66,0.25]	0.28	0.479 [−0.43,1.39]

Note: In all results, tables and figures are the same: *n* = 10. Results are presented as mean ± SE. SE, standard error. C: Control, basal diet; SS: basal diet + 0.3 mg·kg^−1^ sodium selenium; NS: basal diet + 0.3 mg·kg^−1^ nano-selenium; CH: basal diet + 0.3% H_2_O_2_ drinking water; SSH: basal diet + 0.3 mg·kg^−1^ sodium selenium + 0.3% H_2_O_2_ drinking water; NSH: basal diet + 0.3 mg·kg^−1^ nano-selenium + 0.3% H_2_O_2_ drinking water. ^a, b^ Under normal conditions or H_2_O_2_ treatment, different letters indicate significant differences between the different Se source treatments (*p* < 0.05). * Indicates significant difference between H_2_O_2_ treatment and drinking water without H_2_O_2_ (*p* < 0.05). C × SS × NS: *p* value obtained by adding selenium under normal conditions (one-way ANOVA test was performed among the three groups of group C, group SS, and group NS); CH × SSH × NSH: *p* value obtained by adding selenium when feeding H_2_O_2_ drinking water was analyzed (one-way ANOVA test was performed among the three groups of group CH, group SSH, and group NSH); C × CH: the *p* value obtained by feeding H_2_O_2_ without selenium addition was analyzed (*t*-test was performed between the two groups of group C and group CH); SS × SSH: *p* value obtained by feeding H_2_O_2_ when sodium selenite was added (*t*-test was performed between the two groups of group SS and group SSH); NS × NSH: *p* value obtained by feeding H_2_O_2_ when nano-selenium was added (*t*-test was performed between the two groups of group NS and group NSH). *η^2^* is the effect size (eta-squared) of a single-factor variance analysis. *g* is the effect size (Hedges’ g) of the *t*-test analysis. (95% CI) represents the 95% confidence interval of the effect size.

**Table 4:** Effects of nano-selenium on intestinal antioxidation in mice under normal conditions or H_2_O_2_ oxidative stress treatment.

Intestinal Structure	Antioxidant Index	Treatments										
		C	SS		NS		CH		SSH		NSH	
Jejunum	T-SOD (U/mgprot)	504.34 ± 20.92	546.17 ± 27.89		459.99 ± 26.23		490.07 ± 22.43		471.22 ± 35.11		451.84 ± 31.55	
	T-AOC (mmol/g)	0.16 ± 0.01 ^a^	0.17 ± 0.01 ^a^		0.13 ± 0.01 ^b^		0.13 ± 0.00 *		0.14 ± 0.01		0.13 ± 0.01	
	CAT (U/mgprot)	14.00 ± 1.76	14.74 ± 2.30		11.84 ± 1.93		11.57 ± 1.05 ^ab^		13.79 ± 1.84 ^a^		8.56 ± 1.12 ^b^	
	MDA (nmol/mgprot)	0.84 ± 0.07	0.97 ± 0.08		0.71 ± 0.10		0.95 ± 0.08		1.01 ± 0.14		0.80 ± 0.05	
Ileum	T-AOC (mmol/g)	0.15 ± 0.01	0.14 ± 0.01		0.15 ± 0.02		0.14 ± 0.01		0.13 ± 0.02		0.15 ± 0.01	
	MDA (nmol/mgprot)	2.75 ± 0.61	2.62 ± 0.62		2.34 ± 0.36		4.05 ± 0.68		5.76 ± 0.74 *		4.76 ± 1.72	
Colon	T-SOD (U/mgprot)	270.99 ± 9.87 ^b^	333.01 ± 17.33 ^a^		306.79 ± 11.94 ^ab^		277.64 ± 15.22 ^a^		202.51 ± 18.12 ^b^*		251.76 ± 21.25 ^ab^*	
	T-AOC (mmol/g)	0.05 ± 0.00	0.05 ± 0.00		0.05 ± 0.01		0.06 ± 0.01 ^a^		0.04 ± 0.00 ^b^*		0.06 ± 0.00 ^a^	
	MDA (nmol/mgprot)	2.19 ± 0.54	2.78 ± 0.53		3.25 ± 0.89		2.35 ± 0.33		3.03 ± 0.75		2.13 ± 0.49	
			**C × SS × NS**		**CH × SSH × NSH**		**C × CH**		**SS × SSH**		**NS × NSH**	
			*p*-value	*η^2^*-value (95% CI)	*p*-value	*η^2^*-value (95% CI)	*p*-value	*g*-value (95% CI)	*p*-value	*g*-value (95% CI)	*p*-value	*g*-value (95% CI)
Jejunum	T-SOD		0.07	0.181 [0.026,0.47]	0.67	0.031 [0.004,0.35]	0.65	0.204 [−0.72,1.13]	0.11	0.741 [−0.22,1.70]	0.85	0.086 [−0.84,1.01]
	T-AOC		0.01	0.308 [0.087,0.61]	0.80	0.051 [0.003,0.35]	0.01	1.857 [0.77,2.95]	0.06	0.668 [−0.29,1.62]	0.83	−0.096 [−1.02,0.83]
	CAT		0.58	0.040 [0.003,0.34]	0.03	0.249 [0.047,0.60]	0.25	0.509 [−0.41,1.42]	0.76	0.140 [−0.86,1.14]	0.16	0.628 [−0.30,1.55]
	MDA		0.10	0.168 [0.019,0.50]	0.31	0.128 [0.016,0.56]	0.28	−0.473 [−1.39,0.44]	0.78	−0.140 [−1.21,0.93]	0.29	−0.377 [−1.31,0.56]
Ileum	T-AOC		0.88	0.010 [0.002,0.29]	0.71	0.029 [0.002,0.33]	0.21	0.614 [−0.41,1.64]	0.74	0.210 [−0.72,1.14]	0.85	−0.083 [−1.01,0.84]
	MDA		0.96	0.011 [0.003,0.28]	0.41	0.095 [0.010,0.49]	0.16	−0.646 [−1.63,0.34]	0.01	−1.600 [−2.84,−0.36]	0.06	−1.103 [−2.18,−0.03]
Colon	T-SOD		0.02	0.285 [0.064,0.66]	0.02	0.242 [0.081,0.54]	0.88	−0.157 [−1.06,0.74]	<0.01	2.229 [1.06,3.39]	0.03	0.967 [0.01,1.92]
	T-AOC		0.75	0.024 [0.003,0.37]	0.01	0.323 [0.122,0.63]	0.14	−0.683 [−1.64,0.27]	0.03	1.093 [0.06,2.13]	0.41	−0.372 [−1.31,0.56]
	MDA		0.59	0.069 [0.006,0.52]	0.81	0.074 [0.004,0.56]	0.79	−0.147 [1.35,1.06]	0.79	−0.136 [−1.23,0.96]	0.28	0.593 [−0.57,1.76]

## Data Availability

None of the data were deposited in an official repository. The data that support the study findings are available from the authors upon request.
